# *Streptococcus pneumoniae* primary peritonitis mimicking acute appendicitis in an immunocompetent patient: a case report and review of the literature

**DOI:** 10.1186/s13256-019-2038-3

**Published:** 2019-04-28

**Authors:** Francesco Cortese, Pietro Fransvea, Alessandra Saputelli, Milva Ballardini, Daniela Baldini, Aldo Gioffre, Roberto Marcello, Gabriele Sganga

**Affiliations:** 1 Faculty of Medicine and Psychology, University of Rome “La Sapienza”, St. Andrea’s Hospital, Via Di Grottarossa, 1035-39, 00189 Rome, Italy; 2 Emergency Surgery and Trauma Care Unit – St Filippo Neri Hospital, Rome, Italy; 3 Anatomical Pathology – St Filippo Neri Hospital, Rome, Italy; 4 Diagnostic and Interventional Radiology Unit – St Filippo Neri Hospital, Rome, Italy; 5 UOC Chirurgia d’Urgenza, Fondazione Policlinico Universitario Agostino Gemelli IRCCS, Catholic University, Rome, Italy

**Keywords:** Primary peritonitis, *Streptococcus pneumoniae*, Acute abdomen

## Abstract

**Introduction:**

Primary peritonitis without an identifiable intra-abdominal source is extremely rare in healthy individuals; it is commonly seen in cases of nephrotic syndrome, cirrhosis and end-stage liver disease, ascites, immunosuppression, and inflamed peritoneum due to pre-existing autoimmune and oncological conditions.

**Case presentation:**

We present the case of a 68-year-old Caucasian woman operated on due to acute abdomen with a provisional diagnosis of acute appendicitis. During the operation a small amount of free intra-abdominal fluid was found. Her uterus, ovaries, and fallopian tubes were macroscopically normal. Therefore, with the suspicion of appendicitis, appendectomy was performed. Her blood cultures were negative while peritoneal fluid was positive for capsulated form of *Streptococcus pneumoniae*. A 30-day follow-up was performed and she was asymptomatic without any sign of infection.

**Discussion:**

*Streptococcus pneumoniae* commonly causes upper respiratory tract infection and cutaneous infections. It very rarely causes gastrointestinal infection and it is very rarely responsible for primary peritonitis and septic shock syndrome.

**Conclusion:**

Pneumococcal peritonitis has a rare occurrence and represents a clinical challenge because of its subtle and non-specific clinical findings. The interest in our case lays in the relatively rare diagnosis of primary peritonitis mimicking acute appendicitis.

## Introduction

Primary peritonitis (PP) is a diffuse infective inflammation of the peritoneal cavity in the absence of a localized source [1–3]. PP is extremely rare (2%) in healthy individuals; it usually occurs in patients with nephrotic syndrome, cirrhosis, end-stage liver disease, ascites, immunosuppressive status, or inflamed peritoneum due to pre-existing autoimmune and/or oncological conditions [4–7]. The physiopathology of PP is not completely understood, but increased translocation of intestinal bacteria, retrograde diffusion from the genitourinary tract in females, or hematogenous infectious pathways have been discussed. Historically, PP has been related to Gram-negative bacteria, while Gram-positive bacteria, excluding *Enterococcus* species, are rarely involved [8–10] and, therefore, PP was seldom misdiagnosed. We present the case of a 68-year-old Caucasian woman operated on due to acute abdomen with a provisional diagnosis of acute appendicitis.

## Case presentation

**Fig. 1:**
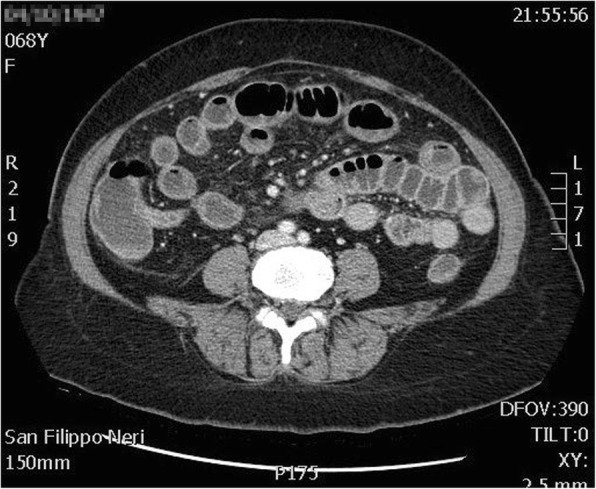
Computed tomography scan image. No abnormalities in the abdominal organs

**Fig. 2:**
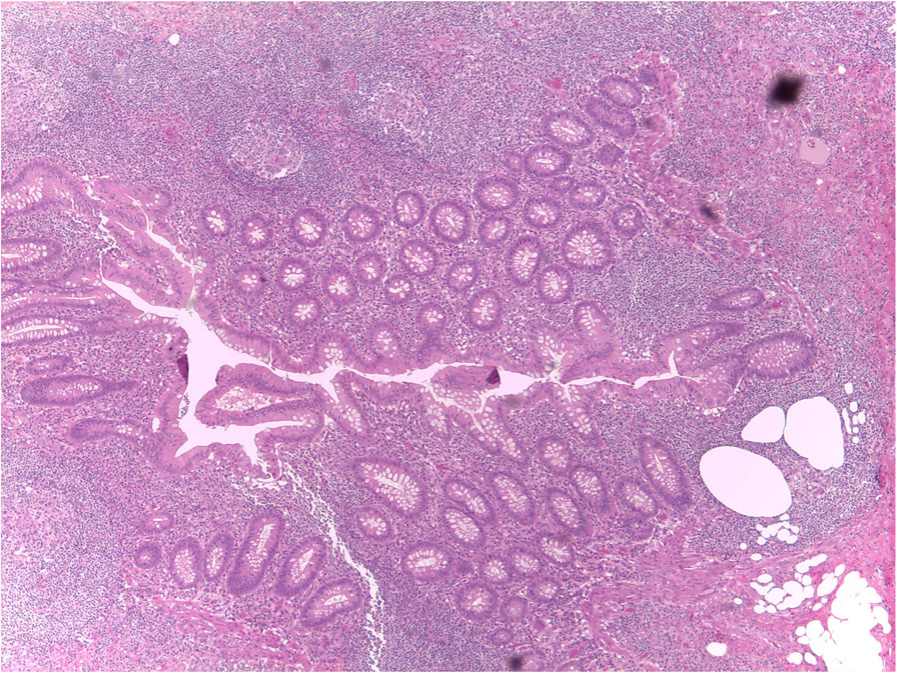
Histopathological examination of the appendix without pathological signs (hematoxylin-eosin, ×10)

**Fig. 3:**
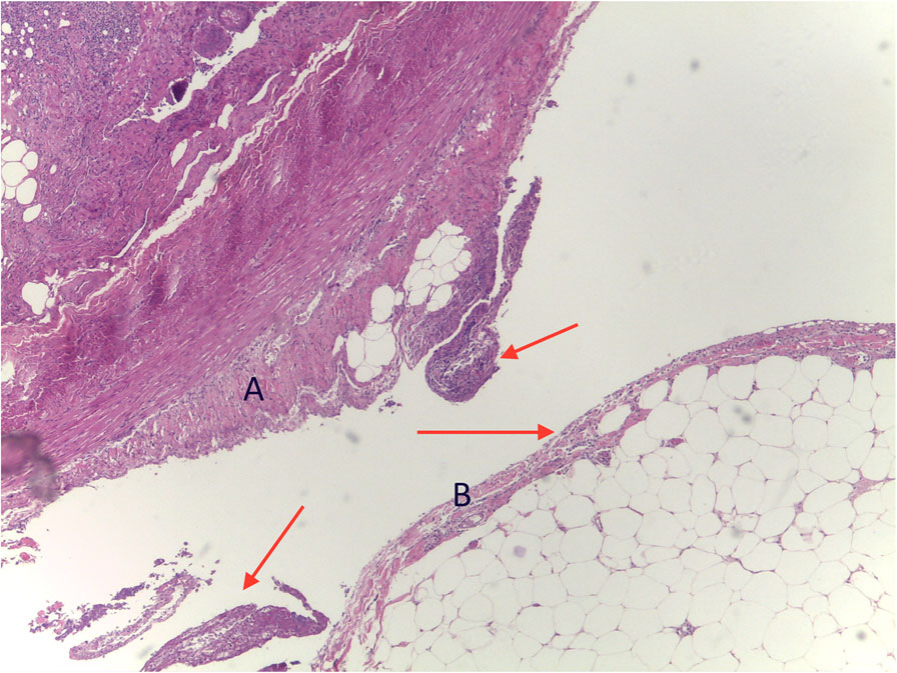
Histopathological report of appendix and mesenteriolum inflammatory peritoneal reaction with deposits of fibrin and granulocytes (*red arrows*) either over appendicular (**a**) and mesenteriolum (**b**) serosa (hematoxylin-eosin, × 10)

A 68-year-old Caucasian woman presented to our emergency department complaining of acute onset of severe abdominal pain in the right lower quadrant that began approximately 48 hours earlier; she had a temperature of 39.1 °C and heart rate of 98/minute. She denied any recent fever, chills, hemoptysis, hematochezia, or change in bowel habits. She had no history of trauma or surgery; she did not take any regular medication; she did not use an intrauterine device (IUD) or other local contraceptive. She had normal sex activity with the same partner (last sexual relationship 20 days before surgery). No relevant history of infection in her family was reported. On her presentation to our emergency room, a physical examination revealed a localized peritonism in the right lower quadrant. At rectal examination, a normal sphincter tone was found with no palpable masses and normal stool. Other features were unremarkable. Laboratory values on admission showed an hemoglobin of 13.3 g/dL, 36.4% hematocrit, with 19.00 × 10^3^/uL white blood cells (WBC). C-reactive protein (CRP) value was 5 mg/dl (normal value < 0.5). A computed tomography (CT) scan (Fig. 1) revealed no pathognomonic signs of appendicitis. Due to the diagnosis of acute abdomen, with provisional clinical diagnosis of acute appendicitis and secondary peritonitis, antibiotic treatment with amoxicillin-clavulanate 2 .2 g three times a day was initiated and she was taken to our operating room. During the operation, a small amount of free intra-abdominal fluid was found with uterus, ovaries, and fallopian tubes being macroscopically normal. Appendicitis was therefore suspected and appendectomy was performed. Ascitic fluid culture was sent to the Microbiology Laboratory in suitable means of transport. The sample was processed with the classic method by sowing on culture-enriched media, searching for aerobic and anaerobic bacteria [11]. *Streptococcus pneumoniae* was isolated after 24 hours of incubation in CO_2_. The organism was identified as *S. pneumoniae* 99.9% with matrix-assisted laser desorption/ionization time-of-flight (MALDI-TOF) mass spectrometry (bioMérieux Clinical Diagnostics). An antibiotic susceptibility test was performed using E-test method and interpreted using European Committee on Antimicrobial Susceptibility Testing (EUCAST) guidelines 2017 [12]. The organism was susceptible to antibiotics tested with minimum inhibitory concentrations (MICs) of benzylpenicillin 0.01 μg/ml, ampicillin 0.02 μg/ml, linezolid 1 μg/ml, ceftriaxone 0.01 μg/ml, meropenem 0.50 μg/ml, levofloxacin 0.5 μg/ml, clindamycin 0.02 μg/ml, trimethoprim/sulfamethoxazole 0.5 μg/ml, and vancomycin 0.1 μg/ml while blood cultures were negative. Our patient’s postoperative course was unremarkable and the antibiotic therapy was stopped after 4 days. She was discharged on the fifth postoperative day asymptomatic with a good performance status. In order to understand the source of this rare form of peritonitis we performed an evaluation of serum oncological markers and immunological status (procalcitonin, interleukin 5, interleukin 10), which were all negative. We also tested markers for HIV and hepatitis C virus (HCV) that gave negative results. A chest CT scan was also performed without any evidence of an active source of infection. Furthermore, histological examination of her appendix did not show signs of appendicitis but revealed a form of peritonitis (Figs. 2 and 3). A 30-day follow-up was performed. At day 10 an evaluation of our patient’s immunological status was performed and the results were negative; at day 20 a chest CT was done and results were negative for any source of infection.

## Discussion

*S. pneumoniae* is the most common cause of community-acquired pneumonia and the second most common cause of purulent meningitis, while intra-abdominal pneumococcal infection is rarely found [13–18].

We conducted a review of the literature by searching the PubMed database for all published series and case reports of PP due to *S. pneumoniae* in the worldwide literature up to 9 September 2017. We analyzed all cases reported in the literature [18–60]. Pediatric cases [19–21], secondary peritonitis [21, 22], and cases arising in patients with ongoing predisposing conditions [23–25] were excluded. All other papers were reviewed in order to evaluate real cases of PP (Table 1) [26, 31, 32, 34–40, 44–51, 53, 54, 56–60]. While pneumococcal peritonitis in children has been recognized for almost 100 years, our review shows, according with the findings of Dugi *et al.* [13], that primary pneumococcal peritonitis without pre-existing peritoneal disease is uncommon in healthy adults [61–64]. Without predisposing conditions, the virulence of some pneumococcus serotypes may contribute to the onset of this rare infection [44, 54]. The physiopathology of primary pneumococcal peritonitis remains controversial. Pneumococci may gain entry to the peritoneal cavity via the genital tract, the gastrointestinal tract, or by hematogenous spread from the respiratory tract [57]. Hemsley and Eykyn [40] reported an increased prevalence in female adults, with the genital tract being the most common source of pneumococcus. In fact, occasionally, vaginal commensals can presumably cause ascending infection also without predisposing factors such as the presence of an IUD or history of recent delivery. However, all the cases reported in their paper have evidence of a presumptive sepsis focus and therefore are not definable as PP.

**Table 1:** Review of the literature

Reference (year)	Author	Age/Gender	Presumed source	Computed tomography	Laparotomy	Peritoneal fluid culture	Blood cultures
[26]/1970	Friedland and Harris	20/F	Unidentified	No	Yes	+	+
[31]/1989	Bukovsky *et al.*	33/F	Unidentified	No	No	–	+
[32]/1989	Davis *et al.*	21/F	Unidentified	No	Yes	+	ND
[34]/1990	Christen *et al.*	50/F	Unidentified	No	Yes	+	+
[34]/1990	Christen *et al.*	58/F	Unidentified	No	Yes	+	ND
[35]/1990	Casadevall *et al.*	87/F	Unidentified	No	Yes	+	+
[35]/1990	Casadevall *et al.*	42/F	Unidentified	Yes	No	+	ND
[36]/1992	Tariq and Joseph	27/F	Unidentified	Yes	No	–	+
[37]/1992	Kunkler *et al.*	36/F	Unidentified	No	Yes	+	–
[37]/1992	Kunkler *et al.*	38/F	Unidentified	No	No	ND	+
[38]/1993	Bruyn	35/F	Unidentified	No	Yes	+	+
[39]/1995	Graham *et al.*	39/F	Unidentified	No	Yes	+	–
[40]/1998	Hemsley and Eykyn	36/F	Unitendified	No	Yes	+	–
[35]/2001	Fox *et al.*	39/F	Unidentified	Yes	Yes	+	ND
[36]/2001	Ueyama *et al.*	52/F	Unidentified	Yes	Yes	+	–
[37]/2001	Sanchez and Lancaster	34/M	Unidentified	Yes	Laparoscopy	+	+
[38]/2004	Okumura *et al.*	29/F	Unidentified	Yes	Yes	+	–
[39]/2004	Kanetake *et al.*	40/M	Unidentified	Yes	Yes	+	–
[40]/2005	Brivet *et al.*	54/F	Unidentified	Yes	Laparoscopy	ND	+
[41]/2005	Brivet *et al.*	82/F	Unidentified	Yes	No	ND	+
[41]/2006	Jarvis *et al.*	38/F	Unidentified	Yes	Yes	+	ND
[42]/2006	Saha *et al.*	23/F	Unidentified	Yes	Yes	ND	+
[44]/2008	Doloy *et al.*	35/F	Unidentified	No	Laparoscopy	+	ND
[45]/2009	Thomas *et al.*	36/M	Unidentified	Yes	Yes	+	+
[47]/2010	Haap *et al.*	27/F	Unidentified	Yes	Yes	ND	+
[48]/2010	Tilanus *et al.*	39/F	Unidentified	Yes	Yes	ND	+
[49]/2010	Monneuse *et al.*	35 (23–43)	Unidentified (4 patients)	Yes	Yes	+	+
[50]/2011	Legras *et al.*	23/F	Unidentified	Yes	Laparoscopy	+	ND
[51]/2013	Malota *et al.*	–	Unidentified (3 patients)	Yes	Yes	+	ND

*F* female, *M* male, *ND* no data

Of interest, a peculiarity of this condition is the absence of mixed organisms. In fact, no case reported an association of multiple infective agents.

There is no definite clinical pattern or features which might help in the diagnosis. Usually the clinical picture closely resembles that of appendicitis or secondary peritonitis with or without sepsis which, in most cases, is the presumptive diagnosis [58–60].

Management of this condition is strictly linked to the diagnosis. While PP can be suspected in patients with comorbidities in the presence of a negative radiologic investigation, it is hardly recognizable in young and immunocompetent patients with no risk factors, like in our case. This confirms the fact that, based on an erroneous diagnosis, most of these patients are operated on despite negative results from imaging.

The best diagnostic algorithm is, in our opinion, Westwood and Roberts’ [63]. Antibiotic therapy remains the first step in treatment of PP in patients with active comorbidities. The real clinical challenge arises with young, healthy, and immunocompetent patients. How can we suspect PP in those cases? In our opinion, with a negative CT scan, antibiotic therapy seems to be the first approach for 36–48 hours, despite several studies underlining that there seems to be little consensus regarding the antibiotic treatment for pneumococcal peritonitis and only little information has been published about the antibiotic regimens chosen for the treatment [65]. For patients infected by penicillin-susceptible organisms, penicillin remains the preferred treatment, while in areas where the prevalence of resistant pneumococci is high, cefotaxime or ceftriaxone is the empirical therapy of choice, as antibiotic-resistant strains of *S. pneumoniae* have been identified worldwide, and the prevalence of these resistant strains is as high as 57% in some countries [61, 64]. In *non-responders* and in patients with sepsis, exploratory laparotomy is a well-accepted treatment [65, 66]. The laparoscopic approach is pivotal, related to its low invasiveness and high diagnostic specificity and sensitivity, with peritoneal lavage or drainage being the diagnostic tool of choice [67]. It remains unclear, however, whether surgical exploration and lavage of the abdominal cavity with or without appendectomy is beneficial or detrimental for patients with primary pneumococcal peritonitis. One could assume that removal of infectious ascites and reduction of intra-abdominal bacterial load would support the healing process, while, in patients with cirrhosis with spontaneous bacterial peritonitis, surgery does not improve the course of the disease [68]. Laparotomy with abdominal debridement and visceral resection is a rare choice selected for advanced cases with complex peritoneal involvement [69, 70]. Based on all these findings it can be assumed that the management of pneumococcal peritonitis involves timely surgical intervention and treatment with antibiotics.

## Conclusion

Primary pneumococcal peritonitis without pre-existing peritoneal disease is rare and represents a cultural and clinical challenge, especially for surgeons, because of its subtle and non-specific clinical findings. The interest in our case lays in the rare diagnosis of PP mimicking acute appendicitis in a healthy woman, without history of recent acute pneumonia or pelvic inflammatory disease (PID) and the isolation of pneumococcus in its capsulated form. This case report reinforces the need for an appropriate clinical algorithm in those patients without medical history and active comorbidities when the diagnosis of acute appendicitis is not clear at the time of operation, especially for female patients. No direct diagnosis is possible. The treatment of choice is the fast initiation of antibiotic therapy. Although surgical therapy is generally not required for the treatment of primary pneumococcal peritonitis, it may be necessary to exclude secondary peritonitis or in non-responders.

## Abbreviations

CRP: C-reactive protein
CT: Computed tomography
EUCAST: European Committee on Antimicrobial Susceptibility Testing
HCV: Hepatitis C virus
IUD: Intrauterine device
MALDI-TOF: Matrix-assisted laser desorption/ionization time-of-flight
MICs: Minimum inhibitory concentrations
PID: Pelvic inflammatory disease
PP: Primary peritonitis
WBC: White blood cells

## References

[1] Koulaouzidis A, Bhat S, Saeed AA. Spontaneous bacterial peritonitis. World J Gastroenterol. 2009;15:1042–9. 10.3748/wjg.15.1042 PMC2655193 19266595

[2] Lata J, Stiburek O, Kopacova M. Spontaneous bacterial peritonitis: a severe complication of liver cirrhosis. World J Gastroenterol. 2009;15:5505–10. 10.3748/wjg.15.5505 PMC2785051 19938187

[3] Laroche M, Harding G. Primary and secondary peritonitis: An update. Eur J Clin Microbiol Infect Dis. 1998;17:542–50. 10.1007/BF01708616 9796651

[4] van Erpecum KJ. Ascites and spontaneous bacterial peritonitis in patients with liver cirrhosis. Scand J Gastroenterol. 2006;243Suppl:79–84. 10.1080/00365520600664342 16782626

[5] Chuang TF, Kao SC, Tsai CJ, Lee CC, *et al.* Spontaneous bacterial peritonitis as the presenting feature in an adult with nephrotic syndrome. Nephrol Dial Transplant. 1999;14:181–2. 10.1093/ndt/14.1.181 10052502

[6] Cheong HS, Joung MK, Kang CI, Ko KS, *et al.* Spontaneous bacterial peritonitis caused by *Streptococcus pneumoniae* in patients with liver cirrhosis. J Inf Secur. 2009;59:218–9. 10.1016/j.jinf.2009.07.006 19656573

[7] Shaw E, Castellote J, Santín M, Xiol X, *et al.* Clinical features and outcome of spontaneous bacterial peritonitis in HIV-infected cirrhotic patients: a case-control study. Eur J Clin Microbiol Infect Dis. 2006;25:291–8. 10.1007/s10096-006-0136-9 16786375

[8] Farthmann EH, Schöffel U. Epidemiology and pathophysiology of intraabdominal infections (IAI). Infection. 1998;26:329–34. 10.1007/BF02962266 9795801

[9] Holzheimer RG, Muhrer KH, L'Allemand N, Schmidt T, *et al.* Intraabdominal infections: classification, mortality, scoring and pathophysiology. Infection. 1991;19:447–52. 10.1007/BF01726463 1816119

[10] Nyström PO, Bax R, Dellinger EP, Dominioni L, *et al.* Proposed definitions for diagnosis, severity scoring, stratification, and outcome for trials on intraabdominal infection. Joint Working Party of SIS North America and Europe. World J Surg. 1990;14:148–58. 10.1007/BF01664867 2183477

[11] Murray PR, Baron EJ, Jorgensen JH, Landry ML, Pfaller MA. Manual of Clinical Microbiology, vol. 1. 9th ed. Washington, DC: ASM Press; 2007.

[12] The European Committee on Antimicrobial Susceptibility Testing. Breakpoint tables for interpretation of MICs and zone diameters, version 7.1, 2017. http://www.eucast.org/fileadmin/src/media/PDFs/EUCAST_files/Breakpoint_tables/v_7.1_Breakpoint_Tables.pdf.

[13] Dugi DD 3rd, Musher DM, Clarridge JE 3rd, Kimbrough R. Intraabdominal infection due to *Streptococcus pneumoniae*. Medicine (Baltimore). 2001;80:236–44. 10.1097/00005792-200107000-00002 11470984

[14] Musher DM. Pneumococcal serotypes and virulence. J Infect Dis. 2006;193:477. author reply 477–8 10.1086/499283 16388500

[15] Watson DA, Musher DM, Jacobson JW, Verhoef J. A brief history of the pneumococcus in biomedical research: a panoply of scientific discovery. Clin Infect Dis. 1993;17:913–24. 10.1093/clinids/17.5.913 8286641

[16] Austrian R. The pneumococcus at the millennium: not down, not out. J Infect Dis. 1999;179(Suppl 2):S338–41. 10.1086/513841 10081505

[17] Musher DM. Infections caused by *Streptococcus pneumoniae*: clinical spectrum, pathogenesis, immunity, and treatment. Clin Infect Dis. 1992;14:801–7. 10.1093/clinids/14.4.801 1576274

[18] Litarski A, Janczak D, Cianciara J, Merenda M. Spontaneous bacterial peritonitis due to streptococcus pneumonia--Case report. Pol Przegl Chir. 2011;83:283–6. 10.2478/v10035-011-0044-2 22166482

[19] Patel RV, Kumar H, More B, Rajimwale A. Primary group A streptococcal septic shock syndrome simulating perforated appendicitis in a previously healthy girl. BMJ Case Rep. 2013;4:2013. 10.1136/bcr-2013-009502 PMC3670001 23645702

[20] Bose B, Keir WR, Godberson CV. Primary pneumococcal peritonitis. Can Med Assoc J. 1974;110:305. PMC1947258 4149584

[21] van Steekelenburg M, de Roo RA, Steenvoorde P, Gosen JJ, *et al.* Pneumococcal peritonitis mimicking acute appendicitis. Eur J Pediatr. 2004;163:505–6. 10.1007/s00431-004-1472-3 15150659

[22] Pasticci MB, Donnini A, Mencacci A, Laparolcia MN, Cavazzoni E, Baldelli F. A diagnosis of pneumococcal peritonitis secondary to pyo-salpinx in a young healthy female by culturing peritoneal pus. New Microbiol. 2008;31:295–8. 18623998

[23] Caierão J, Cornely AF, da Cunha GR, Mott M, *et al. Streptococcus pneumoniae* appendicitis in an adult patient. Am J Emerg Med. 2015;33:990e1–3. 10.1016/j.ajem.2014.12.051 25676850

[24] Kim T, Hong SI, Park SY, Jung J, Chong YP, Kim SH, Lee SO, Kim YS, Woo JH, Lim YS, Sung H, Kim MN, Choi SH. Clinical Features and Outcomes of Spontaneous Bacterial Peritonitis Caused by *Streptococcus pneumoniae*. Medicine (Baltimore). 2016;95(22):e3796. 10.1097/MD.0000000000003796 PMC4900721 27258513

[25] Morrill HJ, Caffrey AR, Noh E, LaPlante KL. Epidemiology of Pneumococcal Disease in a National Cohort of Older Adults. Infect Dis Ther. 2014;3:19–33. 10.1007/s40121-014-0025-y PMC4108120 25134809

[26] Friedland JA, Harris MN. Primary Pneumococcal peritonitis in a young adult. Am J Surg. 1970;119:737–9. 10.1016/0002-9610(70)90251-5 5445999

[27] Herbert TJ, Mortimer PP. Recurrent pneumococcal peritonitis associated with an intra-uterine contraceptive device. Br J Surg. 1974;61:901–2. 10.1002/bjs.1800611110 4434078

[28] Sodhi HS. Primary pneumococcal peritonitis in an adult. J Maine Med Assoc. 1978;69:290–300. 712239

[29] Gruer LD, Collingham KE, Edwards CW. Pneumococcal peritonitis associated with an IUCD [letter]. Lancet. 1983;2:677. 10.1016/s0140-6736(83)92548-5 6136809

[30] Lucas RE, Brook MG. Pneumococcal peritonitis related to an intra-uterine device. J Inf Secur. 1987;15:280–1. 10.1016/s0163-4453(87)92878-7 3693930

[31] Bukovsky I, Neuman R, Ron-El R, Langer R, Caspi F. Pneumococcal peritonitis in the presence of intra-uterine contraceptive device. Conservative treatment: a case report. Eur J Obstet Gynaecol. 1989;33:79–82. 10.1016/0028-2243(89)90081-6 2806711

[32] Davis MG, Halpin DP, O’Byrne P, Stephens RB. Primary pneumococcal peritonitis. A brief report. Ir J Med Sci. 1989;158:307. 10.1007/BF02983694 2628384

[33] Gribbin JC, Cox CJ. Spontaneous bacterial peritonitis in a healthy adult male. Aust N Z J Surg. 1990;60:723–5. 10.1111/j.1445-2197.1990.tb07461.x 2204334

[34] Christen RD, Moser R, Schlup P, Neftel KA. Fulminant group A streptococcal infections: Report of two cases. Klin Wochenschr. 1990;68:427–30. 10.1007/BF01648585 2190045

[35] Casadevall A, Pirofski L, Catalano MT. Primary group streptococcal peritonitis in adults. Am J Med. 1990;88:63N–4N. 2195887

[36] Tariq SM, Joseph TP. Primary pneumococcal peritonitis and bacteremia in an immunocompetent woman [letter]. Clin Infect Dis. 1992;15:375–6. 10.1093/clinids/15.2.375 1520776

[37] Kunkler RB, Grewal HPS, Tomson CRV, O’Brien TS. Primary pneumococcal peritonitis. Br J Hosp Med. 1992;47:262–6. 1591542

[38] Bruyn GAW. Spontaneous pneumococcal peritonitis in young women [letter]. Clin Infect Dis. 1993;16:728–9. 10.1093/clind/16.5.728 8507768

[39] Graham JC, Moss PJ, McKendrick MW. Primary group A streptococcal peritonitis. Scand J Infect Dis. 1995;27:171–2. 10.3109/00365549509019001 7660084

[40] Hemsley C, Eykyn SJ. Pneumococcal peritonitis in previously healthy adults: case report and review. Clin Infect Dis. 1998;27:376–9. 10.1086/514670 9709890

[41] Moskovitz M, Ehrenberg E, Grieco R, *et al.* Primary peritonitis due to group A Streptococcus. J Clin Gastroenterol. 2000;30:325–32. 10.1097/00004836-200004000-00030 10777203

[42] Vuilleumier H, Halkic N. Streptococcal toxic shock syndrome revealed by a peritonitis. Swiss Surg. 2001;7:25–7. 10.1024/1023-9332.7.1.25 11234313

[43] Borgia SM, Low DE, Andrighetti S, Rau NV. Group A streptococcal sepsis secondary to peritonitis and acute pelvic inflammatory disease. Eur J Clin Microbiol Infect Dis. 2001;20:437–9. 10.1007/s100960100511 11476452

[44] Fox KL, Born MW, Cohen MA. Fulminant infection and toxic shock syndrome caused by *Streptococcus pyogenes*. J Emerg Med. 2002;22:357–66. 10.1016/s0736-4679(02)00436-5 12113845

[45] Ueyama N, Kuwashima S, Nakayama A, *et al.* Ileal adenomyoma accompanied by primary peritonitis: Report of a case. Surg Today. 2001;31:826–9. 10.1007/s005950170058 11686566

[46] Sanchez NC, Lancaster BA. A rare case of primary group A streptococcal peritonitis. Am Surg. 2001;67:633–4. 11450776

[47] Okumura K, Schroff R, Campbell R, *et al.* Group A streptococcal puerperal sepsis with retroperitoneal involvement developing in a late postpartum woman: Case report. Am Surg. 2004;70:730–2. 15328810

[48] Kanetake K, Hayashi M, Hino A, *et al.* Primary peritonitis associated with streptococcal toxic shock-like syndrome: Report of a case. Surg Today. 2004;34:1053–6. 10.1007/s00595-004-2863-9 15580392

[49] Brivet FG, Smadja C, Hilbert U, *et al.* Usefulness of abdominal CT scan in severe peritoneal sepsis linked to primary peritonitis. Scand J Infect Dis. 2005;37:76–8. 10.1080/00365540410024899 15764196

[50] Jarvis J, Trivedi S, Sheda S, Frizelle FA. Primary peritonitis in adults: Is it time to look for a better diagnostic classification. ANZJ Surg. 2006;76:127–9. 10.1111/j.1445-2197.2006.03668.x 16626348

[51] Saha P, Morewood T, Naftalin J, Hopkins S. Acute abdomen in a healthy woman: Primary peritonitis due to Group A Streptococcus. J Obstet Gynaecol. 2006;26:700–1. 10.1080/01443610600940232 17071450

[52] van Lelyveld-Haas LE, Dekkers AJ, Postma B, Tjan DH. An unusual cause of a spontaneous bacterial peritonitis in a young healthy woman. N Z Med J. 2008;121:82–5. 18670478

[53] Doloy A, Godin C, Decousser JW, *et al.* Primary peritonitis due to *Streptococcus pyogenes* with reduced susceptibility to fluoroquinolones. Diagn Microbiol Infect Dis. 2008;62:447–9. 10.1016/j.diagmicrobio.2008.08.001 18842379

[54] Thomas D, Perpoint T, Dauwalder O, *et al. In vivo* and *in vitro* detection of a superantigenic toxin Vbeta signature in two forms of streptococcal toxic shock syndrome. Eur J Clin Microbiol Infect Dis. 2009;28:671–6. 10.1007/s10096-008-0671-7 19020908

[55] Kinsella A, Kavanagh DO, McGiobuin S, *et al.* Primary peritonitis from an insect bite. Irish Med J. 2009;102:87–8. 19489198

[56] Tilanus AM, de Geus HR, Rijnders BJ, *et al.* Severe group A streptococcal toxic shock syndrome presenting as primary peritonitis: A case report and brief review of the literature. Int J Infect Dis. 2010;14 Suppl 3:e208–12. 10.1016/j.ijid.2009.07.014 19914115

[57] Monneuse O, Tissot E, Gruner L, *et al.* Diagnosis and treatment of spontaneous group A streptococcal peritonitis. Br J Surg. 2010;97:104–8. 10.1002/bjs.6822 20013929

[58] Haap M, Haas CS, Teichmann R, *et al.* Mystery or misery? Primary group A streptococcal peritonitis in women: Case report. Am J Crit Care. 2010;19:454–8. 10.4037/ajcc2009615 19635804

[59] Legras A, LoDico R, Ferre R, *et al.* Primary peritonitis due to Streptococcus A: Laparoscopic treatment. J Visc Surg. 2011;148:e315–7. 10.1016/j.jviscsurg.2011.07.002 21889922

[60] Malota M, Felbinger TW, Ruppert R, Nüssler NC. Group A Streptococci: A rare and often misdiagnosed cause of spontaneous bacterial peritonitis in adults. Int J Surg Case Rep. 2015;6C:251–5. 10.1016/j.ijscr.2014.10.060 PMC4347958 25555146

[61] Choi SH, Park HG, Jun JB, Lee SO, *et al.* Clinical characteristics and outcomes of pneumococcal bacteremia in adult patients with liver cirrhosis. Diagn Microbiol Infect Dis. 2009;63:160–4. 10.1016/j.diagmicrobio.2008.10.018 19150708

[62] Bucher A, Müller F. Spectrum of abdominal and pelvic infections caused by pneumococci in previously healthy adult women. Eur J Clin Microbiol Infect Dis. 2002;21:474–7. 10.1007/s10096-002-0749-6 12111607

[63] Westwood DA, Roberts RH. Management of primary group A streptococcal peritonitis: a systematic review. Surg Infect. 2013;14:171–6. 10.1089/sur.2012.038 23464678

[64] Capdevila O, Pallares R, Grau I, Tubau F, *et al.* Pneumococcal peritonitis in adult patients: report of 64 cases with special reference to emergence of antibiotic resistance. Arch Intern Med. 2001;161:1742–8. 10.1001/archinte.161.14.1742 11485507

[65] Coialbu T, Minervini F, Pittaluga M, Banderali A, *et al.* Primary pneumococcal peritonitis: description of a case and review of the literature. Clin Ter. 1999;150:449–52. 10756666

[66] Farthmann EH, Schöffel U. Principles and limitations of operative management of intraabdominal infections. World J Surg. 1990;14:210–7. 10.1007/BF01664875 2183484

[67] Farooq A, Ammori BJ. Laparoscopic diagnosis and management of primary bacterial peritonitis. Surg Laparosc Endosc Percutan Tech. 2005;15:36–7. 10.1097/01.sle.0000148464.31652.5d 15714156

[68] Pollock AV. Nonoperative antiinfective treatment of intraabdominal infections. World J Surg. 1990;14:227–30. 10.1007/BF01664877 2183486

[69] Nielsen KR, Ejlertsen T, El-Batran S, Prag J. A five-year survey of pneumococcal peritonitis in two Danish counties. Incidence, diagnosis and clinical entities. Clin Microbiol Infect. 2003;9:738–40. 10.1046/j.1469-0691.2003.00603.x 12925121

[70] El-Samad Y, Fuks D, Lepage L, Hamdad F, *et al.* Treatment in primary *Streptococcus pneumoniae* peritonitis in adult: a case report and review of the literature. Rev Med Intern. 2006;27:966–9. 10.1016/j.revmed.2006.07.008 16997430

